# Factors associated with quality of services for marginalized groups with mental health problems in 14 European countries

**DOI:** 10.1186/1472-6963-14-49

**Published:** 2014-02-03

**Authors:** Diogo Costa, Aleksandra Matanov, Reamonn Canavan, Edina Gabor, Tim Greacen, Petra Vondráčková, Ulrike Kluge, Pablo Nicaise, Jacek Moskalewicz, José Manuel Díaz–Olalla, Christa Straßmayr, Martijn Kikkert, Joaquim JF Soares, Andrea Gaddini, Henrique Barros, Stefan Priebe

**Affiliations:** 1 Department of Clinical Epidemiology, Predictive Medicine and Public Health, University of Porto Medical School, Alameda Prof Hernani Monteiro, 4200-319 Porto, Portugal; 2 Institute of Public Health, University of Porto, Porto, Portugal; 3 Unit for Social and Community Psychiatry, Queen Mary University of London, London, United Kingdom; 4 Health Promotion Research Centre, National University of Ireland Galway, Galway, Ireland; 5 National Institute for Health Development, Budapest, Hungary; 6 Laboratoire de recherche, Etablissement public de santé Maison Blanche, Paris, France; 7 Department of Addictology, First Faculty of Medicine, Charles University in Prague and General University Hospital in Prague, Prague, Czech Republic; 8 Clinic for Psychiatry and Psychotherapy, Charite, University Medicine Berlin, CCM, Berlin, Germany; 9 Institute of Health and Society (IRSS), Université Catholique de Louvain, Bruxelles, Belgium; 10 Institute of Psychiatry and Neurology, Warsaw, Poland; 11 Madrid Salud, Madrid, Spain; 12 Ludwig Boltzmann Institute for Social Psychiatry, Vienna, Austria; 13 Arkin Institute for Mental Health Care, Amsterdam, The Netherlands; 14 Department of Public Health Sciences, Mid Sweden University, Sundsvall, Sweden; 15 Laziosanità ASP–Public Health Agency, Lazio Region, Rome, Italy

**Keywords:** Mental health services, Quality index of service organization, Socially marginalized groups, Multi-level analysis

## Abstract

**Background:**

Different service characteristics are known to influence mental health care delivery. Much less is known about the impact of contextual factors, such as the socioeconomic circumstances, on the provision of care to socially marginalized groups.

The objectives of this work were to assess the organisational characteristics of services providing mental health care for marginalized groups in 14 European capital cities and to explore the associations between organisational quality, service features and country-level characteristics.

**Methods:**

617 services were assessed in two highly deprived areas in 14 European capital cities. A Quality Index of Service Organisation (QISO) was developed and applied across all sites. Service characteristics and country level socioeconomic indicators were tested and related with the Index using linear regressions and random intercept linear models.

**Results:**

The mean (standard deviation) of the QISO score (minimum = 0; maximum = 15) varied from 8.63 (2.23) in Ireland to 12.40 (2.07) in Hungary. The number of different programmes provided was the only service characteristic significantly correlated with the QISO (p < 0.05). The national Gross Domestic Product (GDP) was inversely associated with the QISO. Nearly 15% of the variance of the QISO was attributed to country-level variables, with GDP explaining 12% of this variance.

**Conclusions:**

Socioeconomic contextual factors, in particular the national GDP are likely to influence the organisational quality of services providing mental health care for marginalized groups. Such factors should be considered in international comparative studies. Their significance for different types of services should be explored in further research.

## Background

Risk factors for poor mental health, including social marginalisation, are particularly common in large capital cities [1,2] and these environments deserve more focus in comparative studies on the provision of care for marginalized groups [3]. It has been suggested that comprehensive services addressing a range of different needs might be more efficient in delivering care to marginalized groups with high prevalence of mental disorders, such as the homeless, refugees and asylum seekers, Roma populations, sex workers and the long-term unemployed [4-9].

However, variation in the provision of health services, especially for vulnerable groups, can be attributed not only to the type of clients the units serve but also to the environment or broader context in which service is provided, as reflected in countries’ socioeconomic characteristics [10,11].

The current paper aims to:

describe an Index developed to measure services’ organisation in the context of mental health care provided to socially marginalized people in Europe – the Quality Index of Service Organisation (QISO);

test how the characteristics of services are associated with this created Index;

test how country socioeconomic indicators impact on the Index when comparing European capitals.

## Methods

Good practices in mental health care for socially marginalized groups in Europe were identified through the PROMO project - *Best Practice in Promoting Mental Health in Socially Marginalized People in Europe*[12]. PROMO was designed to assess programmes and systems of services in 14 European countries providing mental health care to socially marginalized groups. Services were assessed in terms of their organisational characteristics, type of clients, components of care and funding arrangements, and how these services interconnect to form systems [12].

The study focused on the following six social groups: the long-term unemployed, the homeless, street sex workers, asylum seekers and refugees, irregular migrants and travelling communities. Data collection was conducted within highly deprived areas of the capital cities of the following 14 European countries: Austria, Belgium, Czech Republic, France, Italy, Germany, Hungary, Ireland, Netherlands, Poland, Portugal, Spain, Sweden and United Kingdom.

A total of 28 highly deprived geographical areas, two in each participating capital city, were identified using local indices of public health and social deprivation. The population size of each area was intended to be between 80,000 and 150,000 inhabitants, with some flexibility to accommodate different local contexts. If chosen areas were too small, they were combined to achieve the target size. The selected areas were: Vienna: District 16 and District 20; Brussels: Schaerbeek & St Josse and Molenbeek; Prague: Prague 3 & 7 and Prague 8; Paris: Secteur Flandre in the 19th arrondissement of Paris and La Courneuve & Aubervilliers in Seine-Saint-Denis; Berlin: Wedding and Kreuzberg; Budapest: District 8 and District 7 & 9; Rome: District 7 and District 15; Dublin: Dublin North Central and Dublin West; Amsterdam: Bos en Lommer & De Baarsjes & Geuzenveld-Slotermeer and Amsterdam Zuid Oost; Warsaw: Praga Polnoc and Wola; Lisbon: Marvila & Santa Maria dos Oliváis and a group of smaller areas (Anjos, Castelo, Encarnação, Graça, Madalena, Mercês, Pena, Penha de França, Santa Catarina, Santa Engrácia, Santa Justa, Santiago, Santo Estêvão, Santos-o-Velho, São Cristóvão e São Lourenço, São José, São Miguel, São Nicolau, São Paulo, São Vicente de Fora, Sé, Socorro); Madrid: Villaverde and Centro; Stockholm: Rinkeby-Kysta & Spånga-Tensta & Skarpnäk and Sodermalm; London: Hackney and Tower Hamlets [13].

The aim was to assess all mental health, social care and general health services that potentially serve marginalized groups with mental health problems. Their organisational characteristics and components, including the type of provider, funding, accessibility, routine data collection, characteristics of staff and programmes provided to people with mental disorders from the marginalized groups were assessed using the *PROMO Tool for Assessment of Services* (available online) [14]. This structured questionnaire was developed through a Delphi process involving experts from the 14 countries. An online platform was developed to facilitate exchange of information amongst participants involved in this process. The final version of the instrument was translated into the languages of participating countries and three pilot interviews were conducted in each capital to assess applicability and suitability.

Data collection was focused on the two identified deprived areas, however, services located outside these areas were also assessed if they were used by clients from the target areas. Available directories and lists were used to identify relevant services, as well as information from local clinicians and experts. Service managers or a member of the staff with relevant knowledge were then contacted via email, telephone or post, and invited to participate after a detailed explanation of the purpose of the study and its implications. They were assessed through face to face or telephone interviews.

Ethical approval was not required for this study, as no patient data were collected.

The services were classified on the basis of their primary focus of care (mental health, general health or social care services) and with regard to the population groups they were serving (either specific to one or more of the PROMO groups or generic, i.e. not focussing on a particular population group). Out of 617 services assessed, 350 were generic services (221 mental health care, 84 social care and 45 general health) and 267 were group-specific services (51 mental health care, 187 social care and 29 general health), (Table 1). Despite the existence of a common protocol for conducting assessments with managers or relevant staff, including numerous reminders for gathering information, some missing information still persisted for variables from all capital cities.

**Table 1:** Typology of services assessed

	**Target population**		**Primary focus of care**		
	**Generic**	**Group-specific**	**Mental health**	**Social care**	**General health**
**Austria**	18 (5.1)	28 (10.5)	9 (3.3)	32 (11.8)	5 (6.8)
**France**	41 (11.7)	21 (7.9)	31 (11.4)	11 (4.1)	20 (27.0)
**Hungary**	4 (1.1)	1 (0.4)	1 (0.4)	1 (0.4)	3 (4.1)
**Poland**	26 (7.4)	16 (6.0)	17 (6.3)	19 (7.0)	6 (8.1)
**Czech Republic**	11 (3.1)	8 (3.0)	6 (2.2)	12 (4.4)	1 (1.4)
**Germany**	79 (22.6)	50 (18.7)	53 (19.5)	66 (24.4)	10 (13.5)
**Italy**	15 (4.3)	19 (7.1)	14 (5.1)	12 (4.4)	8 (10.8)
**Netherlands**	24 (6.9)	13 (4.9)	23 (8.5)	14 (5.2)	0
**Sweden**	0	5 (1.9)	2 (0.7)	0	3 (4.1)
**Belgium**	34 (9.7)	20 (7.5)	21 (7.7)	24 (8.9)	9 (12.2)
**UK**	38 (10.9)	28 (10.5)	40 (14.7)	21 (7.7)	5 (6.8)
**Spain**	6 (1.7)	11 (4.1)	6 (2.2)	11 (4.1)	0
**Portugal**	17 (4.9)	4 (1.5)	13 (4.8)	7 (2.6)	1 (1.4)
**Ireland**	37 (10.6)	43 (16.1)	36 (13.2)	41 (15.1)	3 (4.1)
**Total**	350	267	272	271	74

Figures are n (%).

Services were classified as either generic or group-specific, based on their target users: if more than 50% of the people using a service were from one of the marginalised group, the service was classified as specific for that group.

Social care, mental health or general health service classification was based on service self-definition. In cases where it was not clear whether a service was mental health specific or generic, if 50% of clients were estimated to have a mental health problem the service was classified as a mental health service.

### The Quality Index of Service Organisation score (QISO)

The QISO was developed to facilitate identification of organisational good practice in the context of providing mental health care for socially marginalized people. Its components were defined by the multidisciplinary team of experts involved in the PROMO consortium. The experts’ professional backgrounds were in mental health and social care, public health and social sciences, encompassing both clinical and research expertise. The team of experts discussed and refined each potential quality indicator and its contribution to the overall index score until a consensus was reached on a final set. Evidence generated within the scope of this and other projects in which participating experts were involved was taken into account when developing the QISO [15,16]. This, in turn, resulted in different weightings of each component as a reflection of their importance to the provision of care to marginalised groups. An emphasis was put on self-referrals as the overall service accessibility and networking were highlighted in other PROMO data and in the findings of previous studies on the provision of care in the context of marginalisation. Clinicians working in deprived areas struggle to find adequate services to provide relevant care to the individuals from marginalised groups, with service coordination often being insufficient [13,15]. Amongst the four components of good practice identified in 154 interviews with experts from the 14 capital cities, three directly relate to access and referrals, specifically, facilitating access to services that provide different aspects of health care (reducing the need for further referrals), strengthening the collaboration and co-ordination between different services, and disseminating information on services both to marginalised groups and to practitioners in the area [13].

Therefore, information concerning service organisation comprised indicators covering six domains, with final organisation scores ranging from 0 to a possible maximum score of 15. Quality provision domains and their contribution to the overall score were: accessibility (8), supervision (1), multidisciplinary team (1), programmes provided (2), coordination (1) and evaluation (2).

Quality indicators within each domain correspond to specific service characteristics and account for up to two points of the score as detailed in Table 2. *Accessibility* includes indicators on service opening hours, the existence of exclusion criteria for clients, and accepting self-referrals. *Supervision* refers to the provision of internal or external staff supervision of any type. *Multidisciplinary team* is defined as having staff with both mental health and social care professional backgrounds. *Programmes provided* refers to active outreach programmes and/or home visits to clients as well as case-finding. *Coordination* refers to services having routine meetings with other services. Finally, *Evaluation* includes indicators on recording data on input and attendance, as well as data on client satisfaction.

**Table 2:** Quality Index of Service Organisation–domains, constituting indicators, definition of indicators and their value to the overall score

**Domain**	**Indicator**	**Definition**	**Value**
**Accessibility**	Days open	Open everyday Mon-Fri	1
	Opening hours: *a. Open outside normal office hours*	Open anytime outside normal office hours (Mon-Fri)	1
	Opening hours: *b. Open at weekend*	Open at weekend (anytime)	1
	Exclusion criteria: *a. Lack of motivation*	No to ‘lack of motivation’	1
	Exclusion criteria: *b. Command of language*	No to “command of language of the host country”	1
	Exclusion criteria: *c. Addictions*	No to “addictions”	1
	Self-referrals	Yes to self-referrals	2
**Staff supervision**	Any supervision internal/external	Yes to any supervision (internal/external)	1
**Multidisciplinary team**	Presence of multidisciplinary team	Yes to any combination of mental health and social care professionals (at least one mental health and one social care professional)	1
**Programmes provided**	Active outreach/home visits	Yes to active outreach or home visits	1
	Case finding	Yes to case finding	1
**Coordination**	Routine meetings with other services	Yes to routine meetings	1
**Evaluation**	Recording data on input, attendance and satisfaction	Yes to recording data on input and attendance	1
		Yes to recording outcome data on satisfaction and experience	1

### Service-level variables

In addition to service characteristics, which correspond to the indicators of quality of service organisation, a number of other service features were recorded during the PROMO assessments. In the current analysis, the total number of staff (measured in whole time equivalents, with the number of hours per week defined by each respondent according to his/her national norm) and the number of care programmes provided were used as service-level covariates, due to their importance to the quality of health provision, as asserted in the relevant literature [17], including mental health care studies [18]. Programmes were defined as specific health care or social interventions that each service potentially provides to their clients. Each service was assessed using a specific list of programmes: active outreach, case-finding, home visits, counselling, individual psychotherapy, group psychotherapy, self-help groups, occupational therapy, medication, detoxification and acute withdrawal treatment, drug addiction treatment, alcohol addiction treatment, direct practical help in clients’ homes, befriending, leisure activities support, mental health advocacy, social welfare support, housing/accommodation advice and support, legal advice and support, job coaching/finding, mental health promotion measures and any other programmes specified by the service being assessed.

### Country-level variables

Three Eurostat country-level socioeconomic indicators were included and tested: the country Gross Domestic Product (GDP), the Material Deprivation rate and the Gini coefficient. The GDP is a commonly used measure for assessing a country’s wealth or socioeconomic status, while the Gini coefficient is a measure of income inequality which has been correlated with the prevalence of poor health outcomes and mental disorders [19]. The Material deprivation rate was also chosen because of its direct relevance to the marginalized groups studied, and is considered as an ecological measure of country’s burden of social marginalization [20-22].

The Gross Domestic Product per capita in Purchasing Power Standards (PPS) (2008) has been defined by Eurostat as the value of all goods and services produced less the value of any goods or services used in their creation. The volume index of GDP per capita in Purchasing Power Standards is expressed in relation to the European Union (EU-27) average set to equal 100. A country index higher than 100 corresponds to GDP per capita higher than the EU average. Basic figures are expressed in PPS, a common currency that eliminates differences in price levels between countries, thus allowing meaningful volume comparisons of GDP between countries. This index is intended for cross-country rather than for temporal comparisons.

The Gini coefficient (2008) as a measure of income inequality is conceptualised as the relationship of cumulative shares of the population arranged according to the level of equalized disposable income, to the cumulative share of the equalized total disposable income received by them. The higher the Gini coefficient, the more inequality exists.

The Material Deprivation rate by poverty status (2008) is the percentage of the population with an enforced lack of at least three out of nine material deprivation items depicting material living conditions, such as housing conditions, possession of durables, and capacity to afford basic requirements [23]. The term ‘enforced lack’ refers to people wishing to possess items, but not being able to afford them and the items in question are part of a predefined ‘economic strain and durables’ dimension. ‘Economic strain’ refers to people not being able to afford to do things they would like to do, such as taking a week’s annual holiday away from home, paying a mortgage, rent, utility bills or hire purchase instalments, having a meal with meat, chicken or fish every second day, keeping their home adequately warm, or being able to face unexpected expenses. The durables dimension corresponds to enforced lack of items such as a colour TV, a telephone, a personal car or a washing machine [24].

### Statistical analysis

#### Quality index of service organisation distribution

Descriptive statistics were computed for the QISO distribution across countries. T-tests and ANOVAs were computed to compare and relate types of services with the QISO. An exploratory factor analysis was also performed to test the QISO components and reliability and is presented in Additional file 1.

#### Exploring factors associated with QISO

Correlations between the QISO and the service and country level variables were computed. Unifactorial and multifactorial linear regression analyses were used to examine the association between services characteristics and the QISO.

#### Exploring country differences in QISO

With the linear QISO score as an outcome, four models were built to account for the different levels of the variables: Model 0 (crude) analysed the capital-specific QISO variance without taking into account any other characteristics. Model 1 added the service variables (number of programmes provided and number of staff) in order to understand the role of individual service characteristics in explaining the differences between capital cities. In Model 2, country-level variables including the Gini coefficient and the Material Deprivation Rate were added to the service-level variables and, in Model 3, the GDP was added to models. Country-level effects on the QISO were measured by proportional change in variance from Model 0. Data from Hungary and Sweden were not included in the latter models, as they contributed with too few cases (5 and 2 services respectively).

Interclass Correlation Coefficients (ICC) were computed to show the percentage of observed variation in the QISO that was attributable to capital-level characteristics.

Logistic random effects models were also computed for each domain of the QISO score as outcomes, dichotomized at their median values. Since the outcome constitutes a newly created index, qualitative equal intervals cannot be assumed according to the score variation, requiring this sensitivity analysis. Results of these models are presented in Additional file 1, showing the same change in the ICC from null to fully adjusted models.

Finally, a stratified analysis was performed according to service typology to test differences in terms of the “clients served”. Services were categorised as being either group specific or generic, as well as according to whether they provided mental health care, social care and/or general health care. A *p-*value of <0.05 was considered significant, and only statistically significant service-level variables found in models without stratification were included, together with the country-level variable that meaningfully decreased the ICC.

Analyses were performed using SPSS v.18 (SPSS Inc., Chicago, IL, USA), R v.3.0.0 and Mplus, v. 5.2.

## Results

In the 14 European capitals, 811 services were identified and 617 assessed. In six capitals, less than 70% of services identified were assessed (Prague: 19 services assessed out of 38 identified; Budapest: 5 out of 12; Rome: 34 out of 80; Stockholm: 5 out of 11; Madrid: 17 out of 40 and Lisbon: 21 out of 55).

### Quality Index of Service Organisation (QISO) description

**Figure 1:**
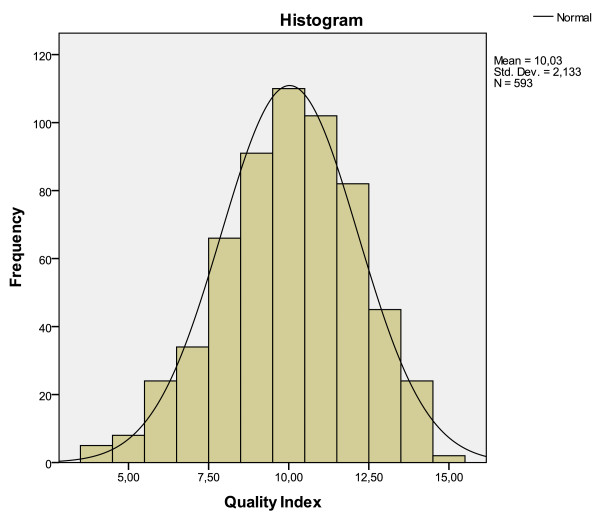
Histogram of Quality Index of Service Organisation score.

The overall QISO was normally distributed, with a mean (SD) of 10.03 (2.13) (Figure 1).

This exploratory factor analysis revealed a 5-factor model as the solution with best fit, generically supporting the theoretical domains for the quality indicators proposed [see Additional file 1].

The mean (SD) number of staff per service across all participating countries was 33.69 (124.28), (Table 3). Services in Hungary had the highest mean (SD) number of staff, 499.20 (885.51), although with very few services contributing to that value. The country with the lowest mean (SD) number of programmes provided per service was Austria with 5.85 (3.00); Poland had the highest with 10.43 (3.84) programmes per service.

**Table 3:** Quality Index of Service Organisation score (QISO) for each country, Number of staff (whole time equivalents), Total programmes provided, Gross Domestic Product (GDP), Gini coefficient and Material Deprivation rate

**Country**	**QISO**		**Staff**		**Total programmes**		**Country GDP**	**Gini coefficient**	**Material deprivation rate**
							**(Eurostat 2008)**	**(Eurostat 2008)**	**(Eurostat 2008)**
	**n**	**Mean (SD)**	**n**	**Mean (SD)**	**n**	**Mean (SD)**			
**Austria**	46	9.11 (1.93)	46	34.06 (77.77)	46	5.85 (3.00)	124	26.2	13.7
**France**	53	10.47 (1.86)	62	38.06 (122.44)	61	7.62 (4.86)	107	29.2	13.1
**Hungary**	5	12.40 (2.07)	5	499.20 (885.51)	5	9.20 (4.21)	64	25.2	37.1
**Poland**	38	10.82 (2.04)	39	41.78 (88.85)	42	10.43 (3.84)	56	32	32.3
**Czech Republic**	19	10.05 (2.27)	19	45.91 (155.13)	19	6.89 (3.71)	81	24.7	16.2
**Germany**	124	9.62 (2.03)	126	10.14 (17.85)	129	8.98 (3.87)	116	30.2	13
**Italy**	32	10.56 (1.63)	34	25.85 (48.93)	34	9.09 (4.00)	104	31	16.1
**Netherlands**	37	9.51 (1.95)	37	17.06 (17.40)	37	10.14 (3.71)	134	27.6	5.2
**Sweden**	2	13.00 (0)	5	10.80 (4.66)	5	9.40 (3.91)	122	24	4.6
**Belgium**	54	10.30 (1.78)	53	23.57 (57.73)	54	8.69 (4.44)	115	27.5	11.6
**UK**	66	11.14 (1.98)	65	20.95 (30.98)	66	9.73 (4.14)	115	33.9	11.3
**Spain**	17	11.35 (1.50)	17	55.76 (93.11)	17	7.06 (4.28)	103	31.3	8.7
**Portugal**	20	11.25 (1.52)	21	90.94 (150.36)	21	9.33 (3.38)	78	35.8	23
**Ireland**	80	8.63 (2.23)	80	11.27 (21.74)	80	7.90 (4.10)	133	29.9	13.6
**Total**	593	10.03 (2.13)	609	29.78 (109.93)	616	8.60 (4.16)			

According to Eurostat for the year 2008, national GDP ranged from 134 for the Netherlands to 56 for Poland. The Gini coefficient varied between 35.8 in Portugal and 24.0 in Sweden. The Material deprivation rate was highest in Hungary (37.1) and Poland (32.3) and lowest in Sweden (4.6) and the Netherlands (5.2).

### Factors associated with QISO

As shown in Table 4, both the number of staff per service, the number of programmes provided per service and the Gini coefficient correlated positively with the QISO across all countries while the GDP correlated negatively. There were no significant differences for mean QISO between generic and specific services: t(591) = -0.77, *p* = 0.44; nor between mental health care, social care and general health care services: F(2,590) = 1.32, *p* = 0.27, nor when considering all six types of services: F(5, 587) = 0.83, *p* = 0.53.

**Table 4:** Correlations and linear regression between the Quality Index of Services Organisation score (QISO) and relevant service-level and country-level variables

	**Total Staff**	**Total Programmes**	**Country GDP**	**Gini coefficient**	**Material Deprivation Rate**
**Spearman’s rho**	0.327*	0.350*	-0.329*	0.220*	-0.066
**Standardized *ß *** **(crude model)**	0.136*	0.352*			
**Standardized *ß *** (adjusted model**)	0.118*	0.348*			

*p < 0.05; Hungary and Sweden not included.

**adjusted model includes both number of staff and number of programmes.

Results from the linear regression models indicated that the number of staff per service and the number of programmes provided per service are significantly associated with the QISO both in the unifactorial analysis and in the multifactorial analysis (*p <* 0.05).

### Country differences in QISO

**Figure 2:**
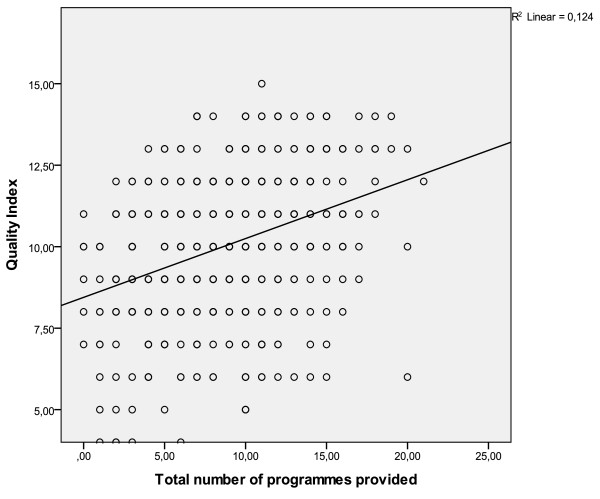
Overall fitted regression line of total number of programmes and QISO score.

**Figure 3:**
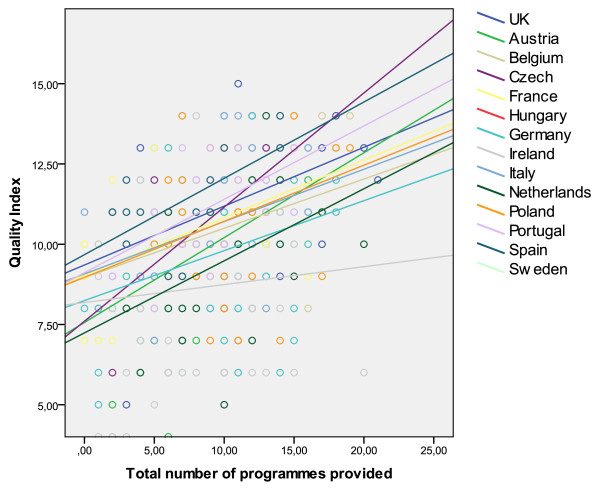
Fitted lines of total number of programmes provided and QISO score by country.

Figure 2 shows the relation of the total number of programmes with the QISO score, in the overall sample. As shown in Figure 3, the intercept and slope of the fitted regression line varies, indicating that the relationship between the QISO and the average number of programmes provided per service varies from one country to the next (Table 4).

As shown in Table 5, in the null random effects model, 14.8% of the variance was explained by country-level traits, as expressed by the Interclass Correlation Coefficient (ICC). The intercept in the empty model was equal to the overall average QISO score, which for this sample was 10.20; the variance component corresponding to the random intercept was 0.67.

**Table 5:** Results from random intercept model for the Quality Index of Service Organisation score–measures of variation

		**Model 0**	**Model 1**	**Model 2**	**Model 3**
	**Fixed**				
	Intercept (SE)	10.20* (0.25)	8.69* (0.28)	5.00 (2.26)	12.69* (2.17)
**Service level**	Total programmes		0.17* (0.02)	0.16* (0.02)	0.16*(0.02)
	Total number of staff		2.10E-3 (1.19E-3)	2.05E-3 (1.09E-3)	1.98E-3 (1.08E-3)
**Country level**	Gini			0.12 (0.08)	0.12 (0.05)
	Material Deprivation rate			-1.88E-3 (0.04)	-0.14 (0.04)
	GDP				-0.05 (0.01)
	**Random**				
	Intercept (SE)	0.67 (0.33)	0.52 (0.26)	0.48 (0.27)	0.10 (0.08)
	Residuals (SE)	3.86* (0.23)	3.42* (0.20)	3.42* (0.20)	3.41* (0.20)
	ICC (%)	14.8	13.2	12.3	2.7

*p < 0.001 (Wald Z statistic for random effects); SE = Standard Error; ICC = Interclass correlation coefficient.

Model 0 = null model, baseline model without any exposure variable.

Model 1 = adjusted for total number of programmes provided by services and number of staff.

Model 2 = additionally adjusted for Gini coefficient (2008) and Material deprivation rate (2008).

Model 2 = additionally adjusted for Gini coefficient (2008), Material deprivation rate (2008) and country-level GDP (2008).

When adding the service-level variables using Model 1 (total number of programmes, total number of staff), the percentage of variance in QISO attributable to country-level variables was 13.2%; in Model 2, the addition of the Gini coefficient and the Material deprivation rate did not represent a marked difference in the ICC (12.3%); only in Model 3 with the addition of the GDP, did the ICC drop to 2.7%, i.e. the country GDP played a role in explaining differences in the QISO independently of the number of staff and programmes, the country Gini coefficient and the Material deprivation rate.

Considering only statistically significant service-level variables (total number of programmes) in the linear regression model, the same proportional decrease in the variance attributable to country-level variables was observed (results not shown).

As shown in Table 6, a decrease in the total variance observed in QISO scores across countries due to country-level variables was observable for all groups of services, although this was more evident for generic services, with a decrease of nearly 8% with the addition of GDP per capita: the ICC dropped from 21.6% to 13.8%. This percentage remained above 20% in the final model for mental health care services and reached 28.0% for general health care services. Only 1.1% of the variance remained attributable to country-level features for social care services from the initial 7.4% in the null model.

**Table 6:** Results from random intercept model for the Quality Index of Service Organisation score–measures of variation, stratified by service target as Group Specific and Generic and by type of care as Mental Health Care, Social Care and General Health Care

		**Service target**		**Service care**		
		**Group specific**	**Generic**	**Mental health**	**Social care**	**General health**
**Model 0**	**Fixed**					
	**Intercept (SE)**	10.06* (0.23)	10.26* (0.32)	10.32* (0.36)	10.03* (0.21)	10.13* (0.46)
	**Random**					
	**Intercept (SE)**	0.40 (0.25)	1.05 (0.53)	1.29 (0.63)	0.31 (0.2)	1.48 (1.18)
	**Residuals (SE)**	3.79* (0.34)	3.83* (0.30)	3.71* (0.33)	3.92* (0.35)	2.61* (0.52)
	**ICC (%)**	9.5	21.6	25.8	7.4	36.2
**Model 1**	**Fixed**					
	**Intercept (SE)**	8.74* (0.34)	8.75* (0.36)	8.58* (0.44)	8.49* (0.31)	9.06* (0.51)
	**Total Programmes**	0.17* (0.03)	0.16* (0.02)	0.17* (0.03)	0.21* (0.04)	0.17 (0.05)
	**Random**					
	**Intercept (SE)**	0.34 (0.22)	0.81 (0.42)	1.11 (0.55)	0.19 (0.16)	0.71 (0.74)
	**Residuals (SE)**	3.48* (0.32)	3.37* (0.27)	3.25* (0.29)	3.49* (0.31)	2.46* (0.50)
	**ICC (%)**	8.9	19.3	25.4	5.0	22.3
**Model 2**	**Fixed**					
	**Intercept (SE)**	10.68* (1.06)	11.27* (1.20)	11.37* (1.48)	10.46* (0.79)	8.97 (2.01)
	**Total Programmes**	0.16* (0.03)	0.16*(0.02)	0.17* (0.03)	0.20* (0.03)	0.16 (0.05)
	**Country-GDP**	-0.02 (0.01)	-0.02 (0.01)	-0.03 (0.01)	-0.02 (0.01)	1.03E-3 (0.02)
	**Random**					
	**Intercept (SE)**	0.23 (0.18)	0.54 (0.32)	0.83 (0.45)	0.04 (0.09)	0.95 (0.94)
	**Residuals (SE)**	3.48* (0.32)	3.37* (0.27)	3.25* (0.29)	3.49* (0.31)	2.44* (0.49)
	**ICC (%)**	6.3	13.8	20.3	1.1	28.0

*p < 0.001 (Wald Z statistic for random effects); SE = Standard Error; ICC = Interclass correlation coefficient.

Model 0 = null model, baseline model without any exposure variable.

Model 1 = adjusted for total number of programmes provided by services.

Model 2 = adjusted for total number of programmes provided by services and country-level GDP (2008).

In summary, country GDP is important for explaining differences in QISO scores across all countries, independently of the number of programmes each service was providing. This remains true regardless of service typology, although the trend is more evident in generic services, i.e. services not specifically focussing on any particular marginalised groups.

## Discussion

A good model fit was obtained for a five-factor model representing the QISO score across countries. The number of programmes provided per service was positively correlated with the QISO score. However, the change in the score related to the increase in the number of programmes varied across countries. Moreover, a decrease was observed in the percentage of QISO score variance attributable to country-level features, mainly with the addition of the GDP estimate. No significant differences were observed in QISO scores when stratifying according to services’ primary target clientele (generic vs. specific) or their primary focus of care (mental health care, social care or general health care).

The decrease in the attributable variance was more pronounced in generic services than in group-specific services and slightly higher in general health services compared to mental health and social care services. More specifically, national GDP matters when explaining QISO score differences between countries, and this remains true independent of the number of programmes that services may be providing. This phenomenon is more apparent in generic services than in group-specific services, and also more apparent in general health care services compared to services focusing on mental health care or social care.

The Quality Index of Service Organization was developed via Delphi process and in-depth discussions among the PROMO team members, representing a large variety of academic and clinical expertise. It reflects elements that were seen as conceptually important for assessing the quality of service organisation in the context of providing mental health care for socially marginalized groups. QISO components were chosen and weighted to match the evidence on health care provision arising from, but not restricted to, the group’s own research [3,12,15,25-27]. The exploratory factor analysis performed (Annex I) confirmed the proposed structure of the QISO domains, although the loadings obtained for the Staff supervision, Multidisciplinary team and Coordination domains were weak, which was expected as they are meant to account individually as distinct constructs with only one item representing each domain.

Nevertheless, the results obtained in the current analysis were further tested with dichotomization of each component and the same changes were observed in hierarchical models, thus strengthening the validity of our measure.

Another strength of the study is the fact that participating services across 14 countries were assessed using a uniform measurement tool with researchers following standardised protocols for interviewing. This is a significant change from traditional approaches to quality of care assessment in the context of marginalisation where objective measures are lacking.

A limitation of this analysis resulted from the absence of reliable and comparable figures describing the size of marginalized groups and the prevalence of mental disorders in each city, which would have allowed testing these associations at a different level. Consequently, the observed associations may be due to other confounding factors that were not accounted for in this analysis [28].

As the number of potential clients is much higher in some countries than in others [29] and the spectrum of mental disorders differs between vulnerable groups [6,7], service development may have been oriented towards different performance targets aiming to provide pragmatic solutions to the daily needs of clients or to comply with vertical governmental policy decisions.

At the service level, the number of programmes was found to be associated with the QISO score, whereas this was not the case for the number of staff members. This suggests that the size of services, in terms of human resources, is less important to organisational quality than the range of approaches provided within each service, which in turn may translate into reduced needs for referrals and thus less expenditure.

In our analysis, the use of the number of programmes as both a constituting domain of the QISO score and as an independent variable in our models could represent some overlap. However, for the QISO domain, only three programmes (out of 22 possible), were considered to count as one point, and the results of the additional sensitivity analysis (Additional file 1) for the dichotomized domains, revealed the same trend in explained variance after addition of service and country level variables, thus favouring our conclusions.

Comparing health services across different countries poses a number of difficulties, including the variability of terminologies employed and arrangements implemented across all types of health services [30]. Our goal was to assess all services that potentially serve individuals from marginalised groups who experience mental health difficulties, and consequently an inclusive understanding of mental health care was applied to accommodate different health and social care systems. As a result, we assessed a variety of services, from large state-funded general hospitals to local and target-specific non-governmental institutions providing care to one of the marginalised groups of interest. Despite the comparison difficulties, we believe the perspective taken is useful for the description of mental health care provision across Europe and for future health policy planning in particular through raising awareness about the number, variety and overlap of different services involved in providing care to marginalised groups and the need for coordination [13].

As previous comparisons of health care provision have shown, contextual factors do matter in delivery of care [10,31]. In a review emphasizing the contribution of epidemiology to government policy, Jenkins [32] showed how representative information collected in a defined geographic area can indicate the actual use of existing services and be utilised to estimate the extent of unmet needs and service provisions required [32]. Research has consistently shown that various measures of social deprivation, positively correlate with psychiatric disorders [33], and that prevalence of these disorders is higher in countries with greater inequality [19].

Given that we assessed services in two highly deprived areas of each capital, homogenous within each city, but heterogeneous between capitals, various characteristics may have influenced the organisational quality of specific services, independently of their nature and the target groups they are serving. However, we observed that simple country-level socioeconomic factors, theoretically close to a broad “deprivation” indicator, seem to influence this relationship.

Probably, the most interesting finding from our work is that the measure of quality organisation in the provision of care is negatively associated with the country GDP. Although the Gini coefficient was not relevant for the country differences, it correlated positively with the QISO score, with which it shows congruency, since “poorer” countries tend to have more inequalities. Mental health care and social care services as well as services targeting specific marginalized groups seemed to be less influenced by this phenomenon compared to generic services and services providing general health care. This suggests that national socioeconomic factors may be more relevant to the quality of care provided by these latter types of services to socially marginalized groups, although the number of programmes provided should also be considered.

The reason why “richer” countries perform less well on the quality score remains unclear. A number of hypotheses could be advanced to explain this phenomenon such as the need of the countries’ to invest in generic health services for this type of population; the fact that having a higher GDP results in relatively smaller numbers of marginalised individuals; or inherent different traditions and investment in social integration compared to services of “poorer” countries where more efforts are made to provide care to marginalized groups. Furthermore, services in countries with higher GDPs may be more efficiently organized, with specific services providing specific programmes, compared to more “disorganized” systems being forced to provide a variety of programmes despite insufficient resources. Finally, it could be argued that richer countries might be providing a greater variety of services, which may result in a more fragmented system as reflected in the QISO.

## Conclusions

In summary, socioeconomic contextual factors, in particular the national GDP, are likely to influence the organisational quality of services providing mental health care for marginalized groups, and this is particularly the case for general health services such as hospitals and primary health care centres, where “poorer” countries perform, on average, slightly “better”. Such factors should be taken into account in international comparative studies of service care provision and in political decision-making related to health care fragmentation and allocation of resources. Their significance for different types of services should also be explored in future research in order to bring further insight into organisational features that might benefit marginalised groups in terms of accessing mental health care. The created QISO score could also be useful beyond the six socially marginalised groups analysed in the scope of the PROMO project, enabling further insight into differences observed between typologies of services (e.g. generic, group-specific), all of which are important for mental health care but often not coordinated, overlapping in their interventions and struggling to overcome known barriers in accessibility.

## Competing interests

The authors declare that they have no competing interests.

## Authors’ contributions

All authors were involved in the research design and data collection procedures. DC performed the statistical analysis for this manuscript and wrote the first draft of the paper. AM, HB and SP, provided further input to the conceptualisation and writing. RC, EG, TG, PV, UK, PN, JM, JMDO, CS, MK, JJFS and AG provided additional revisions to latest versions of the manuscript. All authors contributed to the work, read and agreed to the final manuscript.

## Pre-publication history

The pre-publication history for this paper can be accessed here:

http://www.biomedcentral.com/1472-6963/14/49/prepub

## Supplementary Material

Additional file 1 Results from the exploratory factor analysis performed for the Quality Index of Service Organisation (QISO) and models fitted for QISO dichotomized domains. Click here for file
