# Supplementary material for: Factors associated with quality of services for marginalized groups with mental health problems in 14 European countries
Source: BMC Health Serv Res. 2014 Feb 3;14:49. doi: 10.1186/1472-6963-14-49 (PMC3915221; doi:10.1186/1472-6963-14-49)
Supplement: Additional file 1 — Results from the exploratory factor analysis performed for the Quality Index of Service Organisation (QISO) and models fitted for QISO dichotomized domains. [file 1472-6963-14-49-S1.doc]

**ADDITIONAL FILE 1**

Exploratory factor analysis for the *Quality Index of Service Organisation*.

An exploratory factor analysis (EFA) was performed to test the underlying structure of the created index. The EFA was performed using the maximum likelihood estimation method together with the Geomin rotation. The fit of the QISO was tested using the Tucker-Lewis Index (TLI), the comparative fit index (CFI), the Route mean square error of approximation (RMSEA) and the Standardized root mean square residual (SRMR). The CFI and TFI indexes range from 0 to 1, with higher values indicating a better model fit, whereas to the RMSEA and SRMR, also ranging from 0 to 1, lower values indicate a better model fit. IF CFI and TFI are 0.90 or higher and RMSE and SRMR are close to 0, the model indicates good fit.

Table 1. Factor loadings obtained from the exploratory factor analysis for the Quality Index of Service Organization domains.

|  |  | **Loadings of five-factor solution** | | | | |
| --- | --- | --- | --- | --- | --- | --- |
| **Domain** | **Indicator** | **F1** | **F2** | **F3** | **F4** | **F5** |
| **Accessibility** | Days open | 0.287 | -0.107 | -0.071 | 0.088 | 0.192 |
|  | Opening hours:  *a. Open outside normal office hours* | **0.748** | 0.063 | 0.018 | 0.026 | 0.045 |
|  | Opening hours:  *b. Open at weekend* | **0.993** | 0.000 | -0.010 | -0.027 | -0.148 |
|  | Exclusion criteria:  *a. Lack of motivation* | -0.005 | **0.596** | 0.043 | 0.080 | 0.006 |
|  | Exclusion criteria:  *b. Command of language* | 0.087 | **0.899** | -0.152 | -0.045 | 0.032 |
|  | Exclusion criteria:  *c. Addictions* | -0.150 | **0.588** | 0.136 | 0.074 | -0.190 |
|  | Self-referrals | 0.010 | -0.013 | **0.959** | -0.002 | 0.008 |
| **Staff supervision** | Any supervision internal/external | 0.201 | -0.206 | 0.031 | 0.370 | 0.249 |
| **Multidisciplinary team** | Presence of multidisciplinary team | 0.127 | 0.071 | 0.061 | 0.020 | 0.194 |
| **Programmes provided** | Active outreach/home visits | -0.167 | 0.058 | -0.084 | **0.607** | 0.008 |
|  | Case finding | -0.008 | 0.007 | 0.007 | **0.843** | -0.066 |
| **Coordination** | Routine meetings with other services | 0.069 | 0.042 | 0.112 | 0.291 | 0.129 |
| **Evaluation** | Recording data on input and attendance | -0.020 | 0.049 | 0.200 | -0.090 | **0.717** |
|  | Recording outcome data on satisfaction  and experience | -0.024 | -0.054 | -0.096 | 0.098 | **0.687** |

The Comparative fit index (CFI) was 0.984 and the Tucker-Lewis index TFI was 0.953. The Root mean square error of approximation (RMSEA) was 0.026 and the standardized root mean square residual (SRMR) was 0.040, thus indicating a good global fitness for the QISO.

This exploratory factor analysis supports a five-dimension solution (Table 1). The Staff supervision, Multidisciplinary team and Coordination domains revealed poor loading values amongst the factors. Within the Accessibility domain, the two “Opening hours” items loaded in the first factor and the three “Exclusion criteria” items loaded in the second factor, while the “Self-referrals” item loaded in the third factor. The two items from the Programmes provided domain loaded in the fourth factor while the two items from the Evaluation domain loaded in the fifth factor.

Table 2. Median Odds Ratio (MOR) and Interclass Corelation Coeficients (ICC) from random intercept logistic models

|  |  | **Model 0** | **Model 1** | **Model 2** |
| --- | --- | --- | --- | --- |
| **QISO score** | **MOR** | 1.80 | 1.75 | 1.18 |
|  | **ICC (%)** | 28.10 | 26.15 | 2.78 |
| **Accessibility** | **MOR** | 1.74 | 1.73 | 1.40 |
|  | **ICC (%)** | 25.41 | 25.24 | 11.32 |
| **Staff Supervision** | **MOR** | 1.96 | 2.12 | 1.85 |
|  | **ICC (%)** | 34.38 | 40.26 | 31.08 |
| **Multidisciplinary team** | **MOR** | 1.89 | 1.53 | 1.25 |
|  | **ICC (%)** | 31.38 | 7.56 | 2.77 |
| **Programmes provided** | **MOR** | 1.94 | 2.01 | 1.71 |
|  | **ICC (%)** | 33.16 | 36.43 | 25.12 |
| **Coordination** | **MOR** | 1.80 | 1.75 | 1.37 |
|  | **ICC (%)** | 28.17 | 26.03 | 10.14 |
| **Evaluation** | **MOR** | 1.87 | 1.84 | 1.60 |
|  | **ICC (%)** | 30.58 | 29.26 | 20.01 |

*Domains were dichotomized at their median values;

Model 0 = null model, baseline model without any exposure variable

Model 1= adjusted for total number of programmes provided by services and number of staff

Model 2= additionally adjusted for GINI coefficient (2008) and Material deprivation rate (2008)

Model 2 =additionally adjusted for GINI coefficient (2008), Material deprivation rate (2008) and country-level GDP (2008)
